# MicroRNA in medication related osteonecrosis of the jaw: a review

**DOI:** 10.3389/fphys.2023.1021429

**Published:** 2023-04-26

**Authors:** Siti Salmiah Mohd Yunus, Hui Yuh Soh, Mariati Abdul Rahman, Xin Peng, Chuanbin Guo, Roszalina Ramli

**Affiliations:** ^1^ Department of Oral and Maxillofacial Surgery, Faculty of Dentistry, Universiti Kebangsaan Malaysia, Kuala Lumpur, Malaysia; ^2^ Department of Craniofacial Diagnostics and Biosciences, Faculty of Dentistry, Universiti Kebangsaan Malaysia, Kuala Lumpur, Malaysia; ^3^ Department of Oral and Maxillofacial Surgery, Peking University School of Stomatology, Beijing, China

**Keywords:** microRNA, osteoclast, diagnosis, therapeutics, medication related osteonecrosis of the jaw (MRONJ)

## Abstract

Medication related osteonecrosis of the jaw (MRONJ) is a condition caused by inhibition of the osteoclast activity by the anti-resorptive and anti-angiogenic drugs. Clinically, there is an exposure of the necrotic bone or a fistula which fails to heal for more than 8 weeks. The adjacent soft tissue is inflamed and pus may be present as a result of the secondary infection. To date, there is no consistent biomarker that could aid in the diagnosis of the disease. The aim of this review was to explore the literature on the microRNAs (miRNAs) related to medication related osteonecrosis of the jaw, and to describe the role of each miRNA as a biomarker for diagnostic purpose and others. Its role in therapeutics was also searched. It was shown that miR-21, miR-23a, and miR-145 were significantly different in a study involving multiple myeloma patients as well as in a human-animal study while miR-23a-3p and miR-23b-3p were 12- to 14-fold upregulated compared to the control group in an animal study. The role of the microRNAs in these studies were for diagnostics, predictor of progress of MRONJ and pathogenesis. Apart from its potential diagnostics role, microRNAs have been shown to be bone resorption regulator through miR-21, miR-23a and miR-145 and this could be utilized therapeutically.

## Introduction

Osteonecrosis of the jaw or more commonly known as medication related osteonecrosis (MRONJ) differs from osteonecrosis of other bones in relation to its pathophysiology and treatment. MRONJ occurs as a result of the adverse effect of the antiresorptive or the antiangiogenic drugs (Ruggiero et al., 2014). MRONJ was first documented by Marx, (2003).

The American Association of Oral and Maxillofacial Surgeons Position Paper in 2014 and 2022 (updated version) emphasized on the following “must have criteria” to fulfil the definition of MRONJ (Ruggiero et al., 2014; Ruggiero et al., 2022). The “must have” criteria include.

i. The patients must currently or previously received treatment with antiresorptive or in combination with immune modulators or antiangiogenic agents;
ii. The exposed bone or bone that may be probed through an intraoral or extraoral fistula(e) which has been persisted for more than 8 weeks; and
iii. No history of radiation therapy to the jaws or obvious metastatic disease to the jaws.

The Italian Society for Maxillofacial Surgery (SICMF) and the Italian Society of Oral Pathology Medicine (SIPMO) defined MRONJ as the adverse drug reaction described as the progressive destruction and death of bone that affects the mandible or maxilla of patients who were exposed to the treatment which consist the antiresorptive and antiangiogenic agents and in the absence of previous radiation treatment’ (Bedogni et al., 2012). This definition was affirmed by the Consensus Conference held at the Symposium of the Italian Society of Oral Pathology and Medicine (SIPMO) on 20 October 2018, in Ancona (IT) (Campisi et al., 2020).

In relation to the clinical characteristics, previous literature showed that patients at risk were mostly females, with mean age of 65.3 years and mandible was the most commonly affected jawbone (Cheng et al., 2005; McGowan et al., 2018).

Among the primary diseases reported in the literature, multiple myeloma showed the highest involvement, followed by breast cancer, osteoporosis and prostate cancer (McGowan et al., 2018). Other risk factors are described in the following section.

The clinical appearance of MRONJ can described according to the stage of the disease which were described as Stage 0 to Stage 3 (Ruggiero et al., 2014; Ruggiero et al., 2022).

### Stage of MRONJ

There are three stages of MRONJ (Stage 1–3) (Ruggiero et al., 2014; Ruggiero et al., 2022).

#### Stage 0

Stage 0 is described as a potential precursor of MRONJ as it does not involve an exposed bone or a fistula. The symptoms include odontalgia, jaw pain, which may radiate to the temporomandibular joint region, maxillary sinus pain, or altered sensation of the maxilla and mandibular areas (Ruggiero et al., 2022). There may be presence of an intra or extraoral swelling and mobility of a tooth which could not be associated with periodontal disease. There are changes in the dentoalveolar area which could not be related to any established oral diseases such as loss of the alveolar bone, changes to the trabecular pattern of the sclerotic bone and no new bone formation in the extraction socket, presence of osteosclerosis regions and thickening of the periodontal ligament.

#### Stage 1

There is an exposed necrotic bone or a fistula with no evidence inflammation. The radiographic findings may be similar to Stage 0 and localized to the alveolar bone region.

#### Stage 2

There is an exposed necrotic bone or a fistula with evidence of infection and inflammation these patients are symptomatic. The radiographic findings may be similar to Stage 0 and localized to the alveolar bone region.

#### Stage 3

There is an exposed and necrotic bone or fistulae that probes to the bone, with evidence of infection, and one or more of the complications such as exposed necrotic bone extending beyond the region of the alveolar bone, pathological fracture, extraoral fistula, oral antral or oral-nasal communication or osteolysis extending to the inferior border of the mandible or sinus floor.

Treatment of MRONJ can be either conservative or non-surgical or surgery. Conservative management concentrates on patient education and motivation, reassurance, control of pain and infection (Ruggiero et al., 2022) while surgical intervention involves removing the infected areas and this require margins beyond the borders of the necrotic bone to an area of vital, bleeding bone (Ruggiero et al., 2022). The prognosis of the disease depends on the cancer type and MRONJ among multiple myeloma patients showed poorer healing compared to breast and prostate cancers (Wei et al., 2021).

## Epidemiology of MRONJ

The prevalence of MRONJ among osteoporotic patients on bisphosphonate (BP) was shown to be between 0% and 0.04%, and most reports showed low prevalence of less than 0.001% (Hong et al., 2010; Khan et al., 2011; Urade et al., 2011; Lee et al., 2013). In patients with malignant bone diseases where higher dose of BP was administered, the prevalence was reported to be between 0% and 0.186% (Aragon-Ching et al., 2009; Baqain et al., 2010; Assaf et al., 2013).

The cumulative incidence rate between 2012 and 2014 was shown to be 20.9 per 100,000 person-years (Kim et al., 2021). With regards to denosumab, the incidence of MRONJ ranged from 0 to 30.2 per 100,000 patient-years (Cummings et al., 2009; Orwoll et al., 2012; Papapoulos et al., 2012; Bone et al., 2013).

In relation to route of administration, the incidence of MRONJ ranged from 1.04 to 1.69 per 100,000 patient-years amongst those who were treated with oral antiresorptive agents (Aragon-Ching et al., 2009; Baqain et al., 2010; Fizazi et al., 2011; Hoff et al., 2011; Khan et al., 2011; Urade et al., 2011; Assaf et al., 2013; Lee et al., 2013; Ulmner et al., 2014). The incidence was shown to be higher when intravenous bisphosphonates was administered, i.e., from 0 to 90 per 100,000 patient-years (Lyles et al., 2007; Tennis et al., 2012; Sieber et al., 2013). Regarding the duration of treatment, it was observed that most patients developed MRONJ after receiving BPs for more than a year (Auzina et al., 2019). The incidence was reported to increase to 0.21% if the drugs were consumed for a period of more than 4 years (Pazianas et al., 2007).

## Pathophysiology

MRONJ is a unique type of osteonecrosis which involves the maxilla and mandible (Aghaloo et al., 2015). The paradox of MRONJ pathophysiology led to the following theories, 1) the inhibition of osteoclastic bone resorption and remodelling 2) inhibition of angiogenesis 3) inflammation and infection 4) immune dysfunction 5) soft tissue toxicity (Ruggiero et al., 2014; Aghaloo et al., 2015).

### Theory of altered bone remodelling

Osteoclast is the target cell for the treatment of the metastatic bone malignancy and osteoporosis. The medication, such as the BPs, targets the osteoclast and inhibits its function by specific mechanism, therefore the altered bone remodelling is the fundamental hypothesis for MRONJ (Kimmel, 2007).

The BPs are grouped into 1) non-nitrogen-containing bisphosphonates, for example,: etidronate, clodronate, and tiludronate and 2) nitrogen-containing bisphosphonates such as alendronate, risedronate, ibandronate, pamidronate, and zoledronic acid with different mechanism on the osteoclastic apoptosis pathway. Generally, the BPs exert its effect on the osteoclasts by inhibiting bone calcification and hydroxyapatite breakdown (Drake et al., 2008).

Denosumab, another antiresorptive that is designed to have strong affinity towards receptor activator of nuclear factor-κ signalling/osteoprotegerin (RANK/RANKL/OPG) of the osteoclasts. By binding to this receptor, denosumab blocks the formation and activation of the osteoclasts (Baud’huin et al., 2007). Both denusomab and BPs have detrimental effect on the osteoclasts but in different forms of mechanism. These drugs not only induce osteoclast apoptosis but also affect the cytoskeletal disruption, changing intracellular protein traffic, and blocking the intracellular signal transduction pathways (Reszka & Rodan, 2003). Inhibition of bone remodelling and resorption will then affect the ability of the bone to undergo physiological process of repair following injury. This will consequently lead to necrosis of the bone (Lee et al., 2016).

### Theory of angiogenesis inhibition

Zolendronic acid directly inhibits angiogenesis and together with vascular impairment, this could lead to the development of MRONJ (Chang et al., 2018). The mechanism of action of angiogenesis inhibition is by interfering with the adhesion and migration of human endothelial cells to interrupt tumour invasion and metastases (McLeod et al., 2012). On the other hand, the antiangiogenic medications, such as the VEGF inhibitors, tyrosine kinase receptor inhibitors, and immunomodulatory drugs were also shown to be associated with MRONJ (Gacche & Meshram, 2014; Akita et al., 2018; Vallina et al., 2019). Tyrosine kinase receptor family is involved in tumor growth, pathological angiogenesis and the progression (metastasis) of cancer. Sunitinib inhibits members of the tyrosine kinase receptor family including platelet-derived growth receptors (PDGFRD and PDGFRE), vascular endothelial growth factors (VEGFR1, VEGFR2 and VEGFR3), stem cell factor receptor KIT, tyrosine kinase type 3 (FLT3), the colony stimulating factor 1R (CSF-1R) and the neurotrophic factor receptor derived from the glial cell line (RET) (Hoefert and Eufinger, 2010; Ramírez et al., 2015).

MRONJ was more commonly observed in patients treated in combination between sunitinib and zolendronic acid rather than sunitib alone (Vallina et al., 2019), and MRONJ was more evident with zolendronic acid-sunitinib and not with zolendronic acid and sorafenib, pazopanib, temsirolimus or immunotherapy based on IL-2 (Smidt-Hansen et al., 2013; Ripamonti et al., 2016). This is because sunitinib is more potent against VEGFR and PDGF than the other drugs (Koch et al., 2011; Fusco et al., 2015). In addition, this drug can also cause gingival inflammation and mucositis, delayed wound healing and infection (Vallina et al., 2019).

It is generally accepted that by targeting vascular signalling molecules such as vascular endothelial growth factor (VEGF), the process of tumour invasion and metastases could be inhibited (Zalavras & Lieberman, 2014). However, long term exposure to these agents not only affecting the diseased tissue but also the normal tissue (Zalavras & Lieberman, 2014).

Physiologically, vast formation of new blood vessels will take place after injury in the bone as angiogenesis is the key component of bone repair. During bone repair, a period of necrosis and hypoxia is considered normal phase of healing. New blood vessels bring oxygen and nutrients to the highly metabolically active regenerating callus as well as inflammatory cells, cartilage and bone precursor cells to reach the injury site. However, when this process of vascularization is altered or disrupted, pathologic conditions of healing takes place and this is when necrosis of the bone is inevitable (Lee et al., 2016).

### Theory of inflammation and infection

This theory was initially proposed following reports from several animal studies (Gotcher and Jee, 1981; Mawardi et al., 2009; Aghaloo et al., 2011; Lopez-Jornet et al., 2011; Aguirre et al., 2012; Kang et al., 2013). Animals on antiresorptive treatment that had extraction of the diseased teeth showed impaired healing with mucosal defects, prominent inflammatory infiltrate in the sockets, bone exposure, and areas of osteonecrosis (Aghaloo et al., 2014). Previously, Aghaloo et al. (2011) showed osteocyte necrosis precedes clinical bone exposure in their rat model of BRONJ with aggresive periodontal disease. Extraction of such teeth would injure the surrounding gingival tissues and expose the necrotic bone.

Another study investigated the effect of progressive periodontal disease and MRONJ in the osteoporotic rat which had bisphosphonates intraperitoneally (Li et al., 2016). While alveolar bone resorption from progressive periodontal disease was inhibited, the risk of developing BRONJ was shown to be high (Li et al., 2016).

Hallmer et al. explored the relation between oral flora and MRONJ using the 16S ribosomal RNA pyrosequencing techniques and showed that anaerobic bacteria representative of periodontal microflora, has an effect on the initiation of MRONJ (Hallmer et al., 2017).

Mice that had extractions of healthy teeth exhibited normal mucosal healing and woven bone formation in the sockets. It was also shown that BRONJ did not develop after dental extraction preceded by antibiotic prophylaxis and followed by mucoperiosteal closure in mice receiving bisphosphonates (Abtahi et al., 2013).

In a clinical study that investigated the oral microbiome composition among patients with BRONJ, patients taking bisphosphonates without ONJ, and healthy controls (Kalyan et al., 2015). There was no difference in the oral bacterial community among the groups. Instead, the composition of the oral microbiota across individuals was strongly associated with the function of their systemic leukocytes. Gene expression for RANK, TNF-alpha, and aryl hydrocarbon receptor by peripheral blood leukocytes was found related to the composition of the oral microbiota. These genes expressed by leukocytes are related to immune and stress resiliency (Nguyen et al., 2013; Walsh and Choi, 2014).

### Theory of immune dysfunction

Altered numbers and patterns of T-cells in human and rat MRONJ necrotic bone samples was profound compared to healthy patients and non-MRONJ sites (Qu et al., 2020).

The T cells in the form of alpha beta T cells and gamma delta T cells recognize antigen differently and the role for gamma delta T cells in antimicrobial immunity has been firmly established (Kaufmann, 1996).

The alpha beta T cells were found to stimulate bone loss, while the innate lymphocytes, the gamma delta T cells, were found essential for bone regeneration (Ono et al., 2016). These gamma delta T cells produce interleukin-17A (IL-17A), which promotes bone formation and fracture healing (Kalyan, 2016). The association between MRONJ and deficiency of gamma delta T cells was shown by Kalyan et al. (2013). This study found a significant loss of gamma delta T cells in osteoporotic patients on bisphosphonate therapy. Apart from deficiency of gamma delta T cells, the patients also had underlying conditions that compromised their immune integrity (Kalyan, 2016).

A comprehensive review about the local mucosal and bone immune response in relation to MRONJ can be read from Zhang et al. (2021).

### Theory of soft tissue toxicity

BPs have been shown to be toxic to both oral keratinocytes and fibroblasts, and negatively affect cell cell viability, apoptosis, proliferation and migration (Landesberg et al., 2008; Kobayashi et al., 2010; Cozin et al., 2011; Bullock et al., 2022). During cell proliferation which is crucial wound healing and restoration of the mucosal barrier, keratinocytes were prevented from completing their cell cycles while fibroblasts became apoptotic when treated with BPs (Bullock et al., 2022). In addition, disrupted transforming growth factor beta 1 (TGFβ1) signaling was discovered with delayed periodontal repair in BRONJ human biopsy tissue (Wehrhan et al., 2011). The loss of TGFβ1-related cellular activation in BRONJ-affected oral mucosa connective tissue could describe the delayed wound healing and lack of mucosal regeneration in BRONJ lesions (Wehrhan et al., 2011).

## Risk factors for MRONJ

McGowan et al. (2018) in their systematic review listed the medical and dental risk factors for MRONJ. Dental extraction and periodontal disease were the most commonly reported dental risk factors while chemotherapy, corticosteroids and smoking were the most frequently reported medical risk factors (McGowan et al., 2018). It is the concurrent infection, and not the tooth extraction, that acts as the key risk factor for the development of MRONJ (Otto et al., 2015; Soutome et al., 2018; Hasegawa et al., 2019). In addition, dental extraction with additional osteotomy (Bodem et al., 2015), and root amputation (Hasegawa et al., 2019), increased the risk of MRONJ.

In contrast to the review reported by McGowan et al. (2018), corticosteroids and chemotherapy did not show significant association with the occurrence of MRONJ (Bodem et al., 2015; Hasegawa et al., 2019). Smoking and alcohol tobacco also did not show associations with MRONJ (Yamazaki et al., 2012; Soutome et al., 2018; Hasegawa et al., 2019).

In relation to the medication of concern, higher risk was shown when the medication had been ongoing for 8 months or more (Hasegawa et al., 2019). The risk ratio for high-dose of intravenous medication *versus* low-dose oral administration was 14.6 (95% confidence interval 1.7–125.8) (Yamazaki et al., 2012). Studies also showed that the risk of MRONJ was greater when the anti-angiogenic drugs were combined with the BPs (Christodoulou et al., 2009; Fusco et al., 2015).

## High susceptibility of the jaw bones to MRONJ

The maxillary and mandibular bones originated from the neural crest origin channels (Cheng et al., 2009). The alveolar bone is a part of the maxillary and the mandibular bone and its main function is to support the teeth. In comparison to the limbs and vertebra column, the alveolar bone has a dynamic structure where the bone constantly remodel and adapt to the functional needs (Cheng et al., 2009). Unlike the limbs and vertebral column, the alveolar bone develops as a membrane bone. Significantly, the origin of the alveolar bone influences the phenotype of the osteoblasts and osteoclasts (Kasperk et al., 1995). Osteoblasts from the alveolar bone have an increased rate of cell division in comparison to other bones (Hall, 1999). The behaviour and biochemical structure of osteoclasts originated from alveolar bone are also different from the long bones (Hall, 1999). The overall rate of turnover of the alveolar bone was shown to be ten times greater than that of the long bones (Dixon et al., 1997). The reason for rapid alveolar bone remodeling is mainly due to the mechanical loading stimulus (forces) from mastication (Halldin et al., 2011; Inoue et al., 2019) Bisphosphonates inhibit normal bone remodeling, resulting in the accumulation of microdamage and impaired mechanical properties of the bone (Bertoldo et al., 2007; Weinstein et al., 2009). This decrease in bone remodeling despite the microdamage induced by chewing and local inflammation results in constant exposure of bone to pathogenic microorganisms. These conditions predispose bone tissue to necrosis (Hoefert et al., 2010).

## Dilemma in elucidating MRONJ diagnostic markers

MRONJ is diagnosed clinically when there is an exposure of the bone or fistula in the oral cavity or externally. Precaution could be taken earlier prior to this clinical appearance. Despite several theories describing the possible pathophysiology, at present, there is no specific and reliable diagnostic marker available.

There were many biomarkers that have been proposed to support the diagnosis of MRONJ, for example, Receptor Activator of NF-kB Ligand (RANKL) and osteoprotegerin (OPG) (Bagan et al., 2017), total alkaline phosphatase, bone-specific alkaline phosphatase (BAP), osteocalcin, C-telopeptide (CTX), vascular endothelial growth factor (VEGF), triiodothyronine (T3), thyroxine (T4), thyroid-stimulating hormone (TSH), 25-hydroxyvitamin D, and C-reactive protein (Thumbigere-Math et al., 2016), CTX, undercarboxylated Osteocalcin (Glu-OC), Tartrate-resistant acid phosphatase 5b (TRACP 5b), RANKL, and OPG (Kim et al., 2016) and N-telopeptide (NTX) and BAP (Kolokythas et al., 2015), however, none could provide consistent diagnostic results (Yang et al., 2018). Recently, researchers from Spain showed an important discovery through the proteomic and bioinformatics analyses where three potential protein biomarkers, MMP9, AACT, and HBD were identified (Lorenzo-Pouso et al., 2022).

Novel approach has been proposed to diagnose diseases using circulating microRNAs, which have been shown with high specificity and stability in the body fluids (Turchinovich et al., 2012). MicroRNA biomarkers can be used to indicate presence of a pathology or the stage, progression, or genetic link of pathogenesis (Kocerha et al., 2011; Wang et al., 2011; Weir et al., 2011; Elfimova et al., 2012; Li et al., 2013; Recchioni et al., 2013; Scott et al., 2015; Wang et al., 2015; Biswas, 2018; Hu et al., 2018). The diagnostic performances of the circulating microRNAs have been validated such as in hepatocellular carcinoma (Zhou et al., 2011), heart diseases (Egea et al., 2012) and osteoporosis (Hackl et al., 2016).

In relation to medical intervention, there are some clinical breakthrough reported in various areas. Firstly, Miravirsen, a locked nucleic acid (LNA)-modified antimiR-122 that effectively combats hepatitis C virus infection (Janssen et al., 2013). Secondly, RG-012, an antagomir against miR-21, which reduces fibrogenesis of organs associated with Alport syndrome (Gomez et al., 2015), an X-linked disease which is characterized by hearing loss, visual impairment and kidney disease caused by mutations of the COL4A5 gene (Genetics Home Reference, 2020). Thirdly, Remlarsen or MRG-201, a miRNA mimic that is used for the treatment of pathological cutaneous fibrosis, or other fibrotic diseases, such as idiopathic pulmonary fibrosis (Gallant-Behm et al., 2019). The mechanism of action is by restoring the levels of miR-29b, which is a negative regulator of the extracellular matrix deposition processes (Gallant-Behm et al., 2019). Finally, Cobomarsen (or MRG-106), a LNA antagomir that targets miR-155 which is the focus of treatment for certain types of lymphoma and leukemia (Seto et al., 2018), and Mesomir, a miRNA mimic that could replace miR-16, a tumor suppressor that is reduced in malignant pleural mesothelioma (Reid et al., 2013).

The aim of this review is to describe the role of each miRNA as a biomarker for diagnostic purposes and others. The potentials of miRNAs in therapeutic modalities were also searched.

## MicroRNA

MicroRNAs (miRNAs) are a group of non-protein-coding RNAs comprising 19–25 nucleotides, which are highly preserved during evolution (Parikh et al., 2014). miRNAs control gene expression post-transcriptionally by binding to various mRNA targets (Sotiropoulou et al., 2009).

### MicroRNA and the osteoclasts

The BPs act on the mature osteoclasts by either inactivation or increased apoptosis of the osteoclasts and suppress resorption while denosumab is a fully human monoclonal anti-RANKL antibody that inhibits binding of RANKL to RANK, blocks osteoclast maturation, function and survival, thus reducing bone resorption (Hanley et al., 2012).

miRNAs play a central role in the process of osteoclast growth, differentiation, apoptosis, and bone resorption (Musolino et al., 2018). During normal physiological conditions, osteoclastogenesis is regulated by the osteoblasts and stromal cells, both of which provide two essential factors, macrophage colony-stimulating factor (M-CSF) and receptor activator of nuclear factor NF-ĸB ligand (RANKL) (Ji et al., 2016).

The following miRNAs were expressed in promoting osteoclastogenesis: miR-29, miR-31-5p, hsa-miR-422a, hsa-miR-148a-3p, miR-183-5p, miR-214-3p and miR-9718, while the following miRNAs inhibit osteoclastogenesis: miR-7b-5p, miR-26a-5p, miR-34a-5p, miR-124-3p, miR-125a-5p, miR-146a-5p, miR-218-5p and miR-503-5p (Ge et al., 2017). In addition, three miRNAs were shown with the promotion and inhibition effects: miR-21-5p, hsa-miR-133a-3p and miR-223-3p (Ji et al., 2016) (Table 1).

**TABLE 1:** miRNAs that promote and/or inhibit the osteoclasts.

miRNA	Target genes	Effect on osteoclast
miR-29	CDC42, SRGAP2, NFIA CD93, CALCR	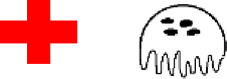
miR-31-5p	RhoA	
hsa-miR-422a	CBL, CD226, IGF1, PAG1, TOB2	
hsa-miR-148a-3p	MAFB	
miR-183-5p	HO-1	
miR-214-3p	Pten	
miR-9718	PIAS3	
miR-21-5p	FasL, PDCD4	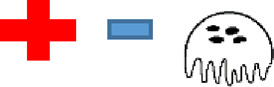
hsa-miR-133a-3p	CXCL11, CXCR3 and SLC39A1	
miR-223-3p	NFIA, IKKα	
miR-7b-5p	DC-STAMP	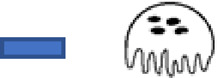
miR-26a-5p	CTGF	
miR-34a-5p	Tgif2	
miR-124-3p	NFATc1, RhoA, Rac1	
miR-125a-5p	TRAF6	
miR-146a-5p	TRAF6, Stat1	
miR-218-5p	p38MAPK-c-Fos-NFATc1	
miR-503-5p	RANK	

hsa: Human; CDC42: Cell Division Control Protein 42; SRGAP2: SLIT-ROBO Rho GTPase-Activating Protein 2; NFIA: Nuclear Factor I/A; CD93: CD93 Molecule; CALCR: calcitonin receptor; RhoA: Ras Homolog Family Member A; CBL: Cbl Proto-Oncogene; CD226:CD226 Molecule; IGF1: Insulin Like Growth Factor 1, PAG1: Phosphoprotein Membrane Anchor with Glycosphingolipid Microdomains 1; TOB2: Transducer Of ERBB2, 2; MAFB: MAF BZIP Transcription Factor B; HO-1: Heme Oxygenase-1; Pten: Phosphatase and Tensin Homolog; PIAS3: Protein Inhibitor of Activated STAT 3; FasL: fas ligand; PDCD4: Programmed Cell Death 4; CXCL11: C-X-C Motif Chemokine Ligand 11; CXCR3: C-X-C Motif Chemokine Receptor 3; SLC39A1: Solute Carrier Family 39 Member 1; NFIA: Nuclear Factor I A; IKKα: Component of Inhibitor of Nuclear Factor Kappa B Kinase Complex; DC-STAMP: dendrocyte expressed seven transmembrane protein; CTGF: connective tissue growth factor; TGIF2: TGFB Induced Factor Homeobox 2; NFATc1: Nuclear Factor of Activated T Cells 1; Rac1: Rac Family Small GTPase 1; TRAF6: TNF Receptor Associated Factor 6; Stat1: Signal Transducer and Activator of Transcription 1; p38MAPK-c-Fos-NFATc1: p38Mitogen-Activated Protein Kinase-Fos Proto-Oncogene- Nuclear Factor Of Activated T Cells 1; RANK: Receptor Activator of NFB.

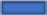
 inhibits

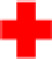
 promotes

### MicroRNA and MRONJ

Evidence of the expression of miRNA and MRONJ can be observed from both human and animal studies. Their characteristics are shown in Table 2.

**TABLE 2:** Summary of the included studies.

Authors, (year published)	Human or animal study	Purpose	Characteristics of the subjects or test animal	Duration of bisphosphonate treatment	Method to induce MRONJ (animal study)	Characteristics of the control	Biological sample used	Results on miRNA
Musolino et al. (2018)	Human	Diagnostic	• 5 multiple myeloma patients with MRONJ (3 female and 2 male)	>1 year		• 5 healthy patients (3 female and 2 male)	Blood	miRNAs that were significantly over-expressed in patients vs. controls
			• median age 61 ± 9 years			• mean age 64 ± 8 years		miR-16-1
			• Location of MRONJ					miR −21
			3 mandible					miR −23A
			2 maxilla					miR −28
								miR -101-1
								miR -124-1
								miR −129
								miR −139
								miR −145
								miR −149
								miR −202
								miR −221
								miR −424
								miR −520
								6 were strongly upregulated (4–11 fold upregulated) in patients vs. controls: miR -16-1
								miR −149
								miR −23A
								miR −145
								miR -129-1
								miR −221
Yang et al. (2018)	Human	• Diagnostic	6 MRONJ patients	No information		11 control patients	Serum	3 miRNA were detected
		• Prediction of progress						2 were elevated in the MRONJ patients compared to the controls. miR-21
								miR-23a
								miR-145 was declined
Yang et al. (2018)	Animal	• Diagnostic	First phase	zoledronate 80 μg/kg body weight injection weekly for 13 weeks	1st and 2nd molars extraction on the left side	First phase	Serum	7 miRNAs were selected from the above study Musolino et al. (2018). miR-21
	• 140 female Sprague-Dawley rats	• Prediction of progress	• 30 rats			• 30 rats		miR-23a
	• 10–12 months old		Second phase			Second phase		miR-28
	• weight: 240–280 g		• 20 rats with 1 week old MRONJ			• 20 rats		miR-124-1
			• 20 rats with 4 weeks old MRONJ			intravenously injected with phosphate-buffered saline		miR-129-1
			• 20 rats with 8 weeks old MRONJ					miR-145
								miR-149
								Comparing the MRONJ rat serum to controls: miR-21 (increased)
								miR-23a (increased)
								miR-145 (decreased)
								miR-28, miR-129-1 and miR-149 (no difference)
								miR-124-1 (could not detected in serum)
Surajkulwatana et al. (2020)	Animal	Pathogenesis	Group 1: 6 rats	administration of zoledronate 66 μg/kg + dexamethasone 5 mg/kg	Maxillary first molar extraction under general anesthesia 2 weeks after first drug administration for all groups	Group 2: 3 rats (Vehicle group) administration of dexamethasone 5 mg/kg	Bone sample from the tooth extraction sites	miRNA increased expression in Group 1 compared to Group 3
	• Twelve Sprague Dawley (SD) rats			Intraperitoneal injection with zolendronic acid and dexamethasone for every 2–3 days for 4 weeks		Group 3: 3 rats administration of normal saline solution		• with 12–14-fold upregulation
	• 6-week-old					Intraperitoneal injection with dexamethasone or normal saline for every 2–3 days for 4 weeks		miR-23a-3p
	• weight range 195–205 g							miR-23b-3p
								• with 5- and 4.5-fold upregulation miR-34a-5p
								miR-24-3p
								• with 5- and 4.5-fold upregulation miR-27a-3p
								miR-652-3p
								• no difference in the expression of miR-663a and miR720 between 2 groups

Abbreviation: MRONJ: medication related osteonecrosis of the jaw.

#### Human studies

There were two studies that used humans as subjects. In the first study, miRNA was investigated in multiple myeloma patients with and without MRONJ. The following miRNAs were significantly over-expressed in MRONJ patients *versus* controls: miR-16-1, miR-21, miR-23a, miR-28, miR-101-1, miR-124-1, miR-129, miR-139, miR-145, miR-149, miR-202, miR-221, miR-424, miR-520. Of the above miRNAs, six were very significantly upregulated in their patients compared to the controls: miR-16-1, miR-149, miR-23a and miR-145, miR-129-1 and miR-221 (Musolino et al., 2018).

The second study also included human subjects concurrently with the animal study. The results were similar to the animals’ study where the level of miR-21 and miR-23a were elevated and miR-145 was declined (Yang et al., 2018).

#### Animal studies

Seven miRNAs (miR-21, miR-23a, miR-28, miR-124-1, miR-129-1, miR-145 and miR-149) from study Muslino et al. (2018) were selected by Yang et al. (2018) to be tested in their animal study. This study aimed to evaluate the diagnostic role and the prediction of MRONJ initiation of the miRNA (Yang et al., 2018). The validation was performed only in the animal study and not for human. They found that the expression of miR-21, miR-23a and miR-145 were significantly different when compared between MRONJ and the controls.

For prediction of MRONJ initiation, only miR-21 could distinguish between one of the MRONJ groups (MRONJ-4 weeks) from the control group. The authors concluded that a combined indice (from miR-21 + miR-23a + miR-145 logistic regression model) was a better diagnostic factor than the individual miRNA and it was also a potential predictor for MRONJ progress.

Another animal study analysed the extraction sockets in zolendronic acid treated rats and controls. This study aimed to investigate the role of miRNA in the pathogenesis of MRONJ. Upregulation of six miRNAs, i.e.,.miR-23a-3p, miR-23b-3p, miR27a-3p and miR-24-3p (these MiRNAs are associated with inhibition of osteoblast maturation) and miR-34-5p and miR-652-3p (anti-angiogenic activity) were shown. The expression of miR-23a-3p and miR-23b-3p were 12- to 14-fold upregulated compared to the control group (Surajkulwatana et al., 2020).

The summary of these studies are shown in Table 2.

The description of miR-21, miR-23a, miR-145 and miR-23b-3p are shown below.

### miR-23a

miR-23a principally targets the interleukins (ILs) (Musolino et al., 2018), tumour necrosis factor, TNF-related targets (Musolino et al., 2018), Fos proto-oncogene (FOS) (Musolino et al., 2018), other genes involved in bone tissue homeostasis such as osteoclast stimulating factor 1 (OSTF1), secreted protein acidic and cysteine rich (SPARC), and SPOCK1 (Musolino et al., 2018) and low density lipoprotein receptor-related protein5 (LRP5) which is closely related with steroid-associated femoral head necrosis (Yang et al., 2018).

miR-23a inhibitor improves osteonecrosis in an animal model (Dong et al., 2017). Selective expression of miR-23a was shown in microvascular endothelial cells *in vivo* (Larsson et al., 2009). miR-23a may have some relation in angiogenesis as it is among the highly expressed miRNAs in the human umbilical vein endothelial cells (Poliseno et al., 2006).

### miR-23b-3p

miR-23b-3p belongs to cluster of miR-23 of the microRNA family, which had been reported to be involved in osteoporosis and in regulation of differentiation of osteoblasts (Zeng et al., 2017; Liu et al., 2018). It was shown that miR-23b-3p regulates differentiation of osteoclasts by targeting the phosphatase and tensin (PTEN) gene (Chai et al., 2019).

### miR-21

miR-21 targets Suppressor of Mothers against Decapentaplegic7 (Smad7), sprouty RTK signaling antagonist 1 (Spry1) and periodontal-ligament-associated protein-1 (PLAP1) to improve osteogenic differentiation (Santini et al., 2003; Oteri et al., 2008; Allegra et al., 2010). miR-21 determines upregulation of osteoclastogenesis via enhancing receptor activator of nuclear factor kappa-Β ligand (RANKL) and suppressing osteoprotegerin (OPG) expression (Alonci et al., 2007). miR-21 is related to particle induced osteolysis pathogenesis (Allegra et al., 2007), and knocking down miR-21 leads to osteoclastogenesis restriction (Katz et al., 2011). Circulating miR-21 is upregulated during MRONJ progress, which is consistent with miR-21 variation in pro-osteoclastogenesis (Yang et al., 2018).

### miR-145

miR-145 principal targets are the ILs (Musolino et al., 2018) and bone homeostasis genes like the syntaxin binding protein 5 like (STXBP5L), osteomodulin, secreted protein acidic and cysteine rich (SPARC), and SPARC/osteonectin, cwcv and kazal-like domains proteoglycan 2 (SPOCK2) (Musolino et al., 2018).

In addition, the highly upregulated miRNAs and their target genes in the human study are shown in Table 3.

**TABLE 3:** The most upregulated miRNAs and their predicted targets (Musolino et al., 2018).

miRNA	Target gene
miR-16-1	• ILs
	• TNFs and TNF-related targets
	o TNFAIP8
	o TNFRSF10
	o TNFRSF10D
	o TNFRSF1A
	• DEAD box helicases (DDXs)
	• X-box binding protein (XBP-related targets)
	o CDH11
	o OMD
	o SPOCK1
	o SPARC
miR-21	• TNFs and TNF-related targets
miR-23A	• ILs
	• TNFs and TNF-related targets
	• DEAD box helicases (DDXs)
	o DDX4
	o DDX50
	o DDX55
	o DDX6
	• X-box binding protein (XBP-related targets)
	o OSTF1
	o SPARC
	o SPOCK1
miR-129-1	• IL6ST
	• DEAD box helicases (DDXs): DDX3X
	• X-box binding protein (XBP-related targets)
	o OMD
	o OSTF1
	o SPOCK2
	o CDH11
miR-145	• ILs
	• TNF and TNF-related targets
	• DDXs
	• X-box binding protein (XBP-related targets)
	o STXBP5L
	o OMD
	o SPARC
	o SPOCK2
miR-149	• IL: IL6 and IL11
	• TNF and TNF-related targets: TNFRSF19 and TNFRSF8
	• X-box binding protein (XBP-related targets)
	o TRAF6
	o TRAF3IP2
miR-221	• IL1RAPL1
	• X-box binding protein (XBP-related targets): OSTM1

Only 4 or less targeted genes are listed here. If there are more than 4 genes involved, only the name of the group is listed, i.e., Interleukins (ILs). ILS: interleukins; TNFs: Tumor Necrosis Factors; TNFAIP8: TNF Alpha Induced Protein 8; TNFRSF10: TNF Receptor Superfamily Member 10a; TNFRSF10D: TNF Receptor Superfamily Member 10d; TNFRSF1A: TNF Receptor Superfamily Member 1a; CDH11: Cadherin 11; OMD: osteomodulin; SPOCK1: SPARC (Osteonectin), Cwcv and Kazal Like Domains Proteoglycan 1; SPARC: secreted protein acidic and cysteine rich; DDX4: DEAD-Box Helicase 4; DDX50: DEAD-Box Helicase 50; DDX55: DEAD-Box Helicase 55; DDX6: DEAD-Box Helicase 6; OSTF1: Osteoclast Stimulating Factor 1; IL6ST: Interleukin 6 Signal Transducer; DDX3X: DEAD-Box Helicase 3 X-Linked; STXBP5L: Syntaxin Binding Protein 5 Like; IL6: Interleukin6; IL11: Interleukin11; TNFRSF19: TNF Receptor Superfamily Member 19; TNFRSF8: TNF Receptor Superfamily Member 8; TRAF6: TNF Receptor-associated Factor 6; TRAF3IP2: TRAF3 Interacting Protein 2; IL1RAPL1: Interleukin 1 Receptor Accessory Protein Like 1; OSTM1: Osteoclastogenesis Associated Transmembrane Protein 1.

Table 3 below shows the miRNAs involved in MRONJ and the target genes in the human study. Only hub genes TNF, interleukin (IL), X-box binding protein (XBP), and DEAD box helicases (DDX) are included in this table as these genes and pathways might have a central action in the progression of MRONJ in multiple myeloma patients (Sun et al., 2016).

## Future clinical use

MRONJ has been the subject of controversy ever since the first case was reported in 2003 (Ruggiero et al., 2014). A multidisciplinary approach plays a fundamental role in the management of this lesion (Ruggiero et al., 2014). Comprehensive dental care prior to commencement of the antiresorptive or antiangiogenic drug treatment considerably reduces the risk of developing MRONJ.

It is widely accepted that miRNAs play a pivotal role in osteogenic differentiation, including osteogenesis and cartilage growth (Maes et al., 2008) and have been shown to control numerous cellular processes such as differentiation, apoptosis, angiogenesis, and cell metabolism (Bartel, 2009).

Based from the literature described above, there are miRNAs that have been identified to be associated with MRONJ. More work is required so that miRNAs can be utilised as 1) a new diagnostic biomarker and 2) potential therapeutic modalities.

### New diagnostic biomarker and therapeutic modality

Emerging evidences showed novel properties of miRNAs as biomarkers in diagnosing MRONJ (Musolino et al., 2018; Yang et al., 2018). Yang et al. (2018) concluded that the altered levels of miR-21, miR-23a and miR-145 were significant between MRONJ and control group and they were detectable as early as 1 week following induction of MRONJ (Yang et al., 2018).

miR-23a and miR-520 were shown under-expressed in patients with multiple myeloma without MRONJ and were over-expressed in MRONJ subjects (Musolino et al., 2018). This miRNAs control cell cycle and angiogenesis and may be used as treatment of MRONJ (Musolino et al., 2018). Blocking the expression of both miRNAs could be the new therapeutic for MRONJ. miR-23A inhibitor has been shown to improve osteonecrosis in an animal model (Dong et al., 2017).

Sun et al. (2016) reported the overexpression of miR-145 during osteoblast differentiation in a human study and Yu et al. (2018) also reported its role in inhibiting RANKL-induced monocytes related osteoclastogenesis in an animal study.

miR-21 was noted to promote osteogenic differentiation by targeting Smad7, Spry1, and PLAP1. miR-21 is also important in upregulating osteoclastogenesis via increasing RANKL and suppressing osteoprotegerin (OPG) expression while the knockdown of miR-21 inhibited osteoclastogenesis. During the progress of MRONJ, the circulating miR-21 was found to be upregulated (Yang et al., 2018).

The above showed a few potential miRNAs that could be utilised for diagnostic tool or therapeutic modality. Although miRNA-related studies in MRONJ are limited, a panel of miRNAs rather than single miRNA could be combined to increase diagnostic sensitivity and/or specificity (Hanna et al., 2019).

## Limitation of the review

There are very few studies that investigate miRNA in relation to MRONJ either as a diagnostic marker or predictor of progress or role in the pathogenesis. Only one study investigated the role of miRNA using human subjects in the primary and validation phase. In addition, the sample size was also small.

## Conclusion

At present, reliable and stable biomarkers for the diagnosis of MRONJ are still far from reality. Although obtaining the miRNA from the body fluid and tissue is extremely technically challenged, successful work on the multipe myeloma patients with MRONJ have been shown. Further exploration into the diagnostic and therapeutic potentials of miR-21, miR-23a, and miR-145, focusing on the reversal of the MRONJ, may produce a novel breakthrough.
